# Fluoroquinolone-based antimicrobial prophylaxis in patients undergoing transrectal ultrasound-guided prostate biopsy

**DOI:** 10.1007/s10096-015-2417-7

**Published:** 2015-06-06

**Authors:** M. Sieczkowski, A. Gibas, M. Bronk, M. Matuszewski

**Affiliations:** Department of Urology, Medical University of Gdańsk, Gdańsk, Poland; Laboratory of Clinical Microbiology, University Clinical Centre in Gdańsk, Gdańsk, Poland

## Abstract

The aim of this study was to establish the prevalence of resistance to fluoroquinolones in *Escherichia coli* strains isolated from patients undergoing transrectal ultrasound-guided prostate biopsy (TRUS-Bx) and to evaluate the incidence of possible infectious complications associated with this procedure. One hundred and four patients undergoing a TRUS-Bx in a single medical centre were prospectively enrolled in this study. In all patients, pre-biopsy rectal swabs were obtained. The analysis determined the antimicrobial susceptibility of *E. coli* strains to levofloxacin, ciprofloxacin and a panel of other antibiotics. Before biopsy, each of the men received a levofloxacin-based prophylaxis. Telephone follow-up was used to identify patients who had complications after TRUS-Bx. Fluoroquinolone-resistant strains were isolated from 9.62 % of the patients. In all cases, there were related to *E. coli* and all those strains were resistant to both levofloxacin and ciprofloxacin. Fluoroquinolones showed greater antimicrobial activity against *E. coli* (*p* < 0.05) than ampicillin, amoxicillin/clavulanate and cephalothin. Minor infectious complications occurred in three patients (2.91 %). The relation between the resistance of *E. coli* to fluoroquinolones and the risk of readmission, as well as infectious complications, was statistically significant (*p* < 0.05). Despite recent reports of increasing prevalence of fluoroquinolone-resistant *E. coli* and the associated increase of severe infectious complications, the presented results have not confirmed this phenomenon. Resistance to fluoroquinolones of *E. coli* strains isolated from rectal swab cultures prior to TRUS-Bx is the risk factor for readmission and infectious complications after this procedure.

## Introduction

Prostate cancer (PCa) is the most common non-skin malignancy among men in developed countries and, due to aging population, its incidence is steadily rising. Furthermore, despite widely discussed overdiagnosis and overtreatment of PCa, it is the second leading cause of cancer-related death in Western men [1–3]. This argues for the need of an effective and safe tool to diagnose this cancer conclusively. At present, systematic transrectal ultrasound-guided prostate biopsy (TRUS-Bx) is the gold standard for histological diagnosis of PCa [4]. TRUS-Bx is considered to be one of the most frequently performed urological procedures in the world. However, due to the procedure of collecting the biopsy samples, this method is fraught with relatively frequent complications [4]. The most common of these conditions are haematuria and haematospermia, which are usually mild and do not require any treatment [4, 5].

Much more significant consequences may result from the infections caused by bacteria originating from the rectal flora. The most common pathogen responsible for them is Gram-negative *Escherichia coli* [6, 7]. In older studies, the incidence of complications like febrile urinary tract infections (UTIs), acute prostatitis or *E. coli* sepsis was low. However, in recent years, many authors have reported a substantial increase of infections in patients following TRUS-Bx [8–10].

Most of these conditions occur despite the use of fluoroquinolones, which are usually the first-line antibiotics in urological prophylaxis. The European Association of Urology (EAU) Guidelines on Prostate Cancer state that they are the drugs of choice prior to TRUS-Bx [4, 11]. Fluoroquinolones is a class of synthetic antimicrobial agents with known effective activity against Gram-negative bacteria. However, many reports have documented the increasing existence of *E. coli* strains resistant to this group of antibiotics [8–10]. This has led to criticism of a currently used antimicrobial prophylaxis [12–16].

Thus, the aim of this study was to establish the prevalence of resistance to fluoroquinolones in *E. coli* strains isolated from patients undergoing TRUS-Bx and to evaluate the incidence of possible infectious complications associated with this procedure.

## Materials and methods

One hundred and four Caucasian males, with a median age of 65 years (range 49–87 years), undergoing a TRUS-Bx in a single medical centre in Gdańsk, Poland, from August 2013 to August 2014, were enrolled in this prospective study.

All study patients had been instructed by the physician regarding possible complications and provided informed consent before prostate biopsy. Indications for TRUS-Bx included an increased serum level of prostate-specific antigen (PSA > 4 ng/ml) and/or abnormal digital rectal examination (DRE). Patients with a previous diagnosis of PCa and/or self-reported allergy to fluoroquinolones were excluded from the study.

The basic data including age, body mass index (BMI), history of previous biopsies and antibiotic therapy (in the 3-month period before TRUS-Bx) were collected.

Subsequently, in all patients, pre-biopsy rectal swabs were obtained using a single cotton-tip applicator with agar gel system (Deltalab, Spain). Within 4 h, samples were transported to the microbiological laboratory and streaked onto Columbia agar (bioMérieux, France) with 5 % sheep blood and MacConkey medium (bioMérieux, France). The plates were incubated aerobically for 24 h at 37 °C. The bacterial strains were identified using the biochemical method in an automated analyser VITEK 2 (bioMérieux, France). The disc diffusion method was used for antibiotic susceptibility testing according to the European Committee on Antimicrobial Susceptibility Testing (EUCAST) criteria [17].

The analysis determined the antimicrobial susceptibility of *E. coli* strains to levofloxacin, ciprofloxacin and a panel of antibiotics, including ampicillin, amoxicillin/clavulanate, cephalothin, cefuroxime, amikacin, trimethoprim/sulphamethoxazole and nitrofurantoin.

Multiple drug resistance (MDR) was defined in this study according to the criteria published by Magiorakos et al. as a non-susceptibility to at least one agent in three or more antimicrobial categories [18].

The results of rectal swab cultures in our study were blinded until the last patient had completed follow-up, with the exception of infectious complications.

After this procedure, each patient received a prescription for a 3-day course of oral levofloxacin (500 mg once a day). All patients were strictly advised to start prophylaxis using the prescribed antibiotic 12 h before TRUS-Bx.

The prostate biopsies were carried out under local anaesthesia of 10 ml 2 % lignocaine. No enema was used and patients were not obligated to fast before this procedure. Biopsies were performed with the patient in the left decubital position using an automated Pro-Mag biopsy gun (Manan Medical Products, USA) with a disposable 16 G biopsy needle (M.D.L., Italy) in conjunction with a medical ultrasound console (BK Medical Ultrasound Scanner 1202 Flex Focus 400, Herlev, Denmark). The specimens were taken from both the lateral and the medial part of the prostate base, midgland and apex. The median biopsy core number was 15 (range 7–16).

After TRUS-Bx, all patients were asked to return to the study centre hospital if they had a fever higher than 38 °C, severe pain, prolongated haematuria or exacerbation of lower urinary tract symptoms. The decision for readmission in each case was considered individually by a urological consultant.

Telephone follow-up with a response rate of 99.04 % was used to identify patients who had complications within 14 days after TRUS-Bx. The follow-up yes-or-no questions were related to complications described in the EAU guidelines assigned to this procedure and included the following symptoms and conditions: haematospermia, haematuria > 1 day, rectal bleeding > 2 days, urinary retention, fever > 38 °C, prostatitis, epididymitis, UTI, urosepsis and other complications requiring hospitalisation or ambulatory treatment [4]. Infectious complications were defined as the secondary conditions that develop after TRUS-Bx and included fever, prostatitis, epididymitis, UTI or urosepsis.

The trial data were entered into a specially designed secure online database accessible only by the project team. The results were analysed with the statistical software MedCalc (MedCalc Software, version 14.8.1, Belgium). Chi-square analysis and Student’s *t*-tests were performed. Statistical significance was defined as *p* < 0.05.

The study was approved by the Local Research Ethics Committee.

## Results

*E. coli* was the most prevalent Gram-negative bacteria, with a presence of 96.15 % of rectal swabs. Fluoroquinolone-resistant strains were isolated from 9.62 % of patients. In all cases, they were related to *E. coli* and all those strains were resistant to both levofloxacin and ciprofloxacin.

The percentage of patients with a previous history of antibiotic treatment within the 3 months before TRUS-Bx tends to be higher (40 %) in the fluoroquinolone-resistant group than in the rest of the patients (13.83 %) (*p* = 0.1102).

**Fig. 1:**
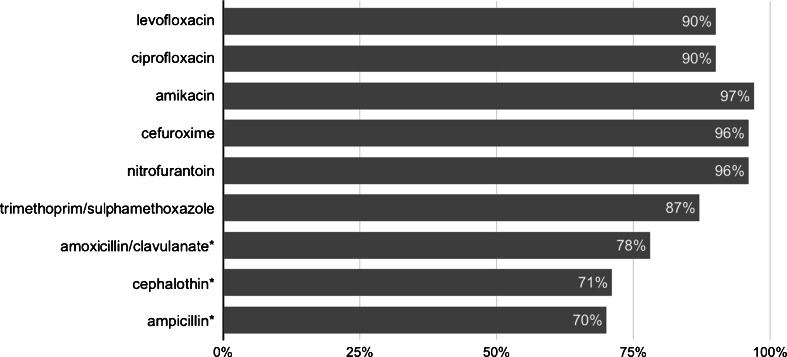
Antibiotic susceptibility of *Escherichia coli* strains. Significant differences between levofloxacin/ciprofloxacin and other antibiotics are marked with an asterisk (*), with *p* < 0.05

Levofloxacin (and ciprofloxacin) showed greater antimicrobial activity against *E. coli* than ampicillin (*p* = 0.0008), amoxicillin/clavulanate (*p* = 0.0339) and cephalothin (*p* = 0.0013, Fig. 1).

**Fig. 2:**
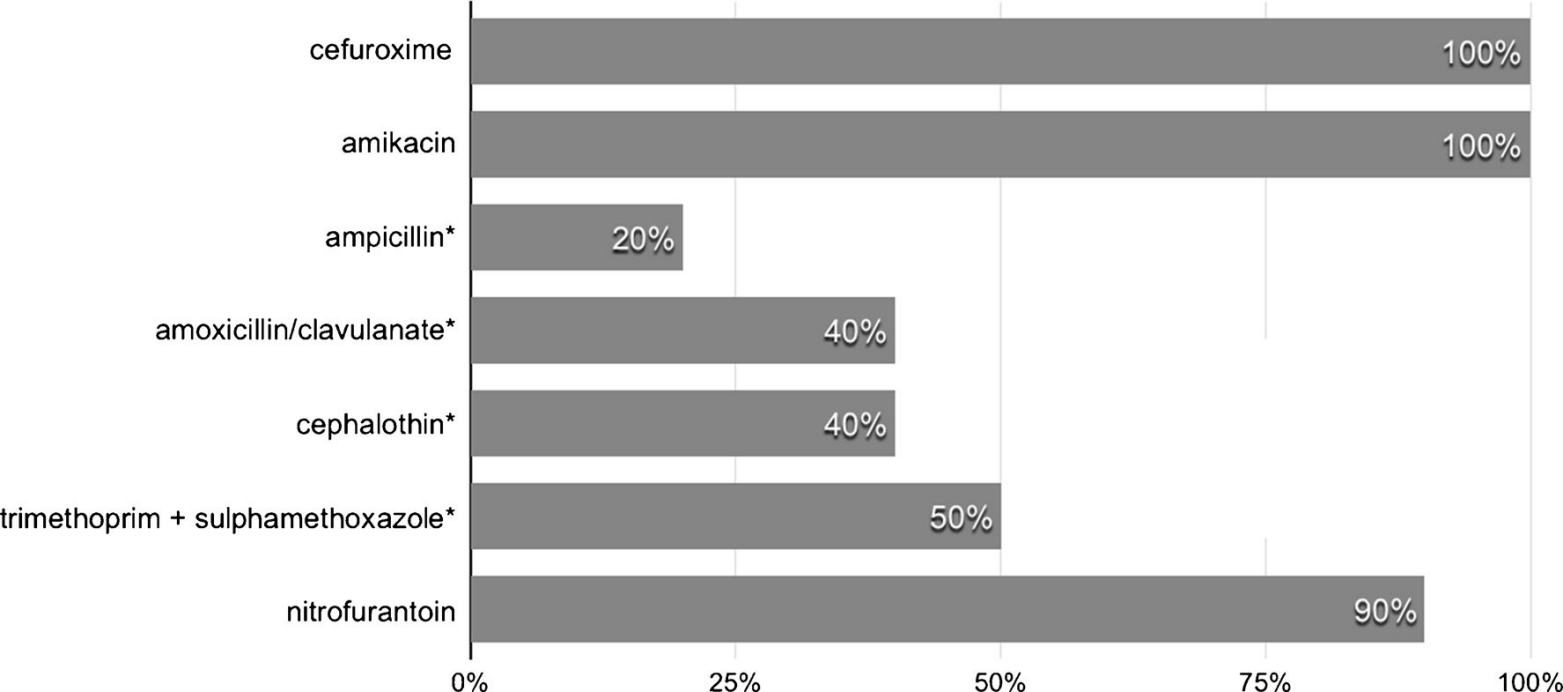
Antibiotic susceptibility of fluoroquinolone-resistant *E. coli* strains. Significant differences between cefuroxime/amikacin and other antibiotics are marked with an asterisk (*), with *p* < 0.05

There were no differences between fluoroquinolones and trimethoprim/sulphamethoxazole, amikacin or nitrofurantoin on this point. All fluoroquinolone-resistant strains of *E. coli* were susceptible to amikacin and cefuroxime (Fig. 2). The relation between the resistance of *E. coli* to fluoroquinolones and the risk of readmission (*p* = 0.0123), as well as infectious complications (*p* = 0.0168), was statistically significant. There was no correlation between fluoroquinolone resistance and other complications, like haematuria and haematospermia (Table 1).

**Table 1:** Summary of the clinical data according to the fluoroquinolone resistance of *Escherichia coli* strains

Data	Fluoroquinolone-resistant *E. coli* (+)	Fluoroquinolone-resistant *E. coli* (−)	Total
Number of patients	10 (9.62 %)	94 (90.38 %)	104 (100 %)
Average age	68.5	66.99	67.13
Average BMI	27.25	27.54	27.47
Antibiotics 3 months before TRUS-Bx	40 %	13.83 %	16.35 %
Previous TRUS-Bx	30 %	11.7 %	13.46 %
Average IPSS before TRUS-Bx	13.80	12.31	12.45
MDR*	60 %	5.32 %	10.58 %
Average prostate volume (ml)	40	46.66	46.02
Median PSA (ng/ml)	9.12	7.91	7.93
Readmission rate*	20 %	1.08 %	2.91 %
Infectious complications*	20 %	1.08 %	2.91 %
Haematuria	30 %	47.31 %	45.63 %
Haematospermia	0	16.12 %	14.56 %

*BMI* body mass index; *TRUS-Bx* transrectal ultrasound-guided prostate biopsy; *IPSS* International Prostate Symptom Score; *MDR* multiple drug resistance; *PSA* prostate-specific antigen

Significant differences between groups are marked with an asterisk (*), with *p* < 0.05

In 10.58 % of all patients, we observed MDR *E. coli* strains (60 % of which were resistant to fluoroquinolones). The presence of MDR strains was significantly associated with the risk of rehospitalisation (*p* < 0.0001), as well as the occurrence of infectious complications (*p* = 0.0042).

The most common complications after TRUS-Bx in the study group were prolonged haematuria (>1 day) (45.63 %) and haematospermia (14.56 %). Infectious complications occurred in three patients (2.91 %). Two of them had a fluoroquinolone-resistant *E. coli* strain (Table 1).

Three of the patients required a short-term rehospitalisation: two of them due to UTI with fever and one because of congenital coagulopathy and haematuria. There was no urosepsis after TRUS-Bx in the studied group.

The history of previous TRUS-Bx and the number of biopsy cores taken had no influence on the incidence rate of complications.

## Discussion

According to the EAU guidelines, oral or intravenous fluoroquinolones are the state-of-the-art prophylaxis prior to prostate biopsy [4, 11]. Due to the transrectal access used during the biopsy, fluoroquinolones seem to be an effective option, as their spectrum covers Gram-negative bacteria. Several recent studies have underlined the growing incidence of fluoroquinolones resistance that may lead to increased risk of infectious complications after prostate biopsy. It is postulated that the increased resistance to these antibiotics is related to the widespread use of fluoroquinolones in everyday medical practice [7].

We observe that a significant number of patients undergoing TRUS-Bx had a previous history of antibiotic treatment for various reasons. What is more, prescribing antibiotics for men with a newly elevated PSA level, on the presumption that the patient has prostatitis, is still a common urological practice [19].

This phenomenon seems to be confirmed by the results of our study, where 16.35 % of participants were taking antibiotics within the 3 months before biopsy. In the group of patients with *E. coli* strains resistant to fluoroquinolones, this percentage was even more than three times as great as in the remaining group (40 % vs. 13.83 %, Table 1). The results are consistent with the publication by Steensels et al., who showed that receiving fluoroquinolones before biopsy significantly increased the risk of rectal colonisation by these strains [15].

Rectal swab cultures analysis has shown that at least 90 % of *E. coli* strains were sensitive to amikacin, cefuroxime, nitrofurantoin and fluoroquinolones (levofloxacin and ciprofloxacin) (Fig. 1). The results of our study do not support other reports with a large percentage of *E. coli* strains resistant to fluoroquinolones in patients undergoing TRUS-Bx. In relation to the data published by Liss et al. (18 %, California, USA), S Taylor et al. (19.0 %, Vancouver, Canada), AK Taylor et al. (19.6 %, Chicago, USA), Steensels et al. (22 %, Leuven, Belgium) and Lee at al. (26.7 %, Jeonbuk Province, Korea), our results are more than twice as low (9.62 %) [15, 20–23]. Only the results reported by Batura et al. (10.6 % London, UK) appear comparable [24]. This indicates a significant geographical variation of fluoroquinolone-resistant *E. coli* strains. This issue has also been discussed in the review by Erb et al. in symptomatic patients, who noted a higher prevalence of those strains in Latin American countries and Spain than in patients from North America, Central Europe and the British Isles [25].

Furthermore, all of the *E. coli* strains isolated from our studied population demonstrated simultaneous insensitivity to ciprofloxacin and levofloxacin. *E. coli* may acquire resistance for both antibiotics in the same way. This appears to be the result of one of three main mechanisms: development of efflux (pumping drugs out of the cell), alterations in the fluoroquinolone enzymatic targets (e.g. DNA gyrase, topoisomerase IV) or decreased outer membrane permeability. Therefore, a selective resistance to one of these antibiotics is rare [26].

Another problem in the acquisition of resistance by *E. coli* is MDR. Those strains have become a major public health issue in many countries and the main reason for treatment failure of UTI [27]. Our results confirmed that the presence of MDR *E. coli* strains in faecal carriage is a risk factor for readmission after TRUS-Bx due to infectious complications.

The case–control study by Carignan et al. showed a significantly increased rate of infectious complications after prostate biopsy, from 0.52 % in 2002–2009 to 2.15 % in 2010–2011, despite prophylaxis [16]. In 2006, Davidson et al. first reported MDR *E. coli*-related sepsis following transrectal prostate biopsy [28]. Since then, many researchers have been trying to find alternative methods of prostate cancer diagnosis. One of the possibilities is transperineal biopsy, which still has some limitations, including: increased time, need for longer training and financial constraints, as well as the need for general or spinal anaesthesia. Furthermore, the necessary equipment for transperineal biopsy is not widely available. That makes it a marginally performed procedure in comparison with TRUS-Bx [29].

The increase of prostate biopsy-related infectious complications also justifies the need to search for new effective prevention schemes. A number of antibiotic prophylaxis modifications have been proposed. These include the use of other fluoroquinolones (from second and third generation) in various oral and intravenous doses, changes of antibiotic classes (like aminoglycosides, sulfonamides or even carbapenems) or different lengths of prophylaxis [13, 30, 31]. However, none of these methods has solved the issue with prostate biopsy-related infections.

One of the potential ways to decrease TRUS-Bx-related infectious complications is to use targeted antimicrobial prophylaxis. Performing rectal swabs culture prior TRUS-Bx can identify carriers of *E. coli* strains resistant to fluoroquinolones and choose proper prophylactic antibiotics [21]. In 2010, Batura et al. reported a strong correlation between the antimicrobial susceptibility of rectal swabs collected before TRUS-Bx and cultures from blood and urine [24]. These cultures provide useful data for selecting appropriate antibiotics for prophylaxis and treatment of infections associated with prostate biopsy.

All the patients in our study received levofloxacin prior to TRUS-Bx, besides the results of rectal swab cultures. This enabled to determine the effectiveness of currently used routine regimens of antimicrobial prophylaxis.

The group of patients with rectal flora resistant to fluoroquinolones showed a significantly higher risk of developing infectious complications and readmission (Table 1). However, the potential benefit of targeted antibiotic prophylaxis could refer only to 1 of 52 men who underwent rectal swab culture before prostate biopsy. The result is even less favourable than that published by Taylor et al. (1/38) and is probably related to lower rates of resistance to fluoroquinolones [21]. This rate may grow along with the increase in the incidence of MDR *E. coli* strains in the population. It should also be taken into account that the rectal swab cultures do not always correspond to the pathogen isolated from urine (or blood) in the case of infectious complications. In our group, this situation occurred in 1 of 3 patients who developed infectious complications. Kim et al. showed that all four of their patients who developed fever after TRUS-Bx had fluoroquinolone-sensitive rectal flora [32]. Furthermore, none of the patients presented in this study (233 men) developed urosepsis. The authors explained it by the potentially small study group. This could also be the reason for the absence of such severe complications in our study; the average number of patients developing urosepsis after TRUS-Bx despite prophylaxis was between 0.3 and 2.8 %. Another reason could be the smaller number of fluoroquinolone-resistant *E. coli* strains in our group.

Our data showed a similar level of other minor infectious complications (2.91 %) in comparison with previous studies [5].

Despite the fact that the most common complications after TRUS-Bx were haematuria (45.63 %) and haematospermia (14.56 %), they usually did not require any treatment. It was the infectious complications that were the most common reason for readmission after TRUS-Bx.

Considering the number of prostate biopsies taken worldwide, the burden of the management of infectious complications is very significant. Exploring possible influencing factors is very important. We have confirmed that there was neither correlation between infectious complications and the numbers of cores collected during prostate biopsy nor the history of previous TRUS-Bx. Steensels et al. reported a similar conclusion that repeat biopsy alone is not a risk factor for the faecal carriage of fluoroquinolone-resistant strains [15]. However, the influence of the number of biopsy cores collected during prostate biopsy on developing complications seems to be controversial. There are some reports describing the number of biopsy cores as a risk factor influencing the development of infectious complications, as well as haematuria, haematospermia and rectal bleeding [33–37], whereas the majority of studies did not detect an association between complication rates and this factor [38–44].

Several limitations need to be considered in discussing the results of this study. Follow-up contact was limited, for the most part, to telephone interviews. This could be a potential source of bias in the interpretation of post-biopsy complications, even though the response rate of telephone follow-up was high.

Another limitation of the study was the omission of feverishness/low-grade fever (37–38 °C) in the telephone follow-up questions. Consideration of this condition, which may potentially be the cause of the patient’s discomfort, could possibly increase the complications rate. However, low-grade fever would hardly be recognised as an infectious complication. Non-infectious minor post-operative complications appear to cause feverishness, whereas higher temperature values usually concern an infectious cause.

## Conclusions

Despite the limitations, this study shows that resistance to fluoroquinolones and multiple drug resistance (MDR) of *Escherichia coli* strains isolated from rectal swab cultures prior to transrectal ultrasound-guided prostate biopsy (TRUS-Bx) are the risk factors for readmission and infectious complications after this procedure. Rectal swab cultures can identify bacterial strains which are a potential source of infectious complications after prostate biopsy and may be used for the planning of a targeted and empiric antimicrobial prophylaxis.

However, the results of this research has, so far, not confirmed the recent phenomenon of increasing prevalence of fluoroquinolone-resistant *E. coli* strains and the associated increase of severe infectious complications.
